# Improved Functional Outcomes Following Operative Treatment of Midshaft Clavicle Fractures in an Active Duty Population

**DOI:** 10.7759/cureus.7488

**Published:** 2020-03-31

**Authors:** Nicholas Lake, Kyle W Mombell, Ethan Bernstein, Kevin O'Mary, Jasmine Scott, Bradley Deafenbaugh

**Affiliations:** 1 Orthopaedic Surgery, Naval Medical Center San Diego, San Diego, USA; 2 Orthopaedics and Rehabilitation, University of Texas Medical Branch, Galveston, USA; 3 Orthopaedics, Naval Medical Center San Diego, San Diego, USA

**Keywords:** clavicle, fracture, midshaft, functional, outcomes, operative, military

## Abstract

Clavicle fractures are common orthopedic injuries that occur in a young active population and are even more common in the military. Military fitness test data presents the unique opportunity to analyze functional ability with regard to military-specific activities. The primary goal of this study was to compare functional outcomes using military fitness test data between operative and non-operative treatment of midshaft clavicle fractures.

We performed a retrospective review of active-duty U.S. Navy and Marine patients with midshaft clavicle fractures treated at our institution over a seven-year period. There were 94 and 153 patients in our operative and non-operative cohorts, respectively. Average follow-up time from the date of injury or surgery was 28 months.

The rate of infection in the operative group (4%) was significantly greater than in the non-operative group (0%, *p* = 0.023). The rate of non-union in the operative group (3%) was significantly lower than in the non-operative group (14.5%, *p* = 0.004). The rate of symptomatic malunion in the operative group (0%) was significantly different from that in the non-operative group (4.6%, *p* = 0.036). There was no significant difference in the rate of revision surgery between the operative (9.2%) and non-operative (13.2%) groups (*p* = 0.105).

A total of 51 marines met inclusion criteria for our functional outcome analysis using Marine Corps Physical Fitness Test (PFT) data. Of those who underwent operative fixation, 68% were able to meet or surpass their pre-injury average amount of pull-ups in their first PFT after surgery and 88% by the next PFT at least one year after surgery. While 69% of non-operative patients met their pre-injury average in their first PFT, only 57% maintained this level at least one year after surgery. This difference was statistically significant.

In our functional outcome subgroup analysis, we found improved outcomes for pull-ups at least one year out when midshaft clavicle fractures are treated operatively compared to non-operatively. While similar findings in the literature are based on functional outcome questionnaires, physical fitness performance data has not been reported on to our knowledge.

## Introduction

Clavicle fractures are common orthopedic injuries that occur in a young active population and account for 2.6% of all fractures [1]. They are more common in the military than in the general population, with an incidence of nearly one per 1000 persons annually [2]. Peak incidence is seen in patients in their second decade and decreases with increasing age. The majority of these injuries occur in the midshaft of the bone [3]. Traditionally, midshaft clavicle fractures were treated non-operatively with reportedly excellent results and low rates of nonunion [4]. More recent studies, including level one clinical trials, have supported surgical management of clavicle fractures to decrease nonunion and symptomatic malunion rates [5-6]. Specifically, fixation of those fractures that have radiographic characteristics of complete displacement, shortening, and comminution has been proposed. Specific patient populations, including overhead laborers, competitive athletes and smokers have also been described in the literature as benefiting from operative fixation [7-11]. While the functional outcomes of operatively versus non-operatively treated injuries have been traditionally considered similar, there is recent data to suggest improved functional scores in surgically treated patients. This data has consisted of improved shoulder endurance as well as improved Disability of the Arm, Shoulder, and Hand (DASH) scores in operatively treated injuries [12]. However, these outcomes have been measured via questionnaires rather than physical tests. Military fitness test data presents the unique opportunity to analyze functional ability with regard to military-specific activities. The primary goal of this study was to compare functional outcomes using military fitness test data between operative and non-operative treatment of these injuries. As the physical demands of active duty military differ from the general population, we believe this functional data will be more applicable to the military patient. In addition, we evaluated complications and additional surgeries and lost work time in both treatment groups. This information will allow us to better counsel patients on their return to military-specific duties and their ability to pass their service-specific physical fitness tests. We hypothesized that operatively treated clavicle fractures would more quickly return to baseline function than their non-operatively treated counterparts, though they will ultimately have a similar return to function in the long term.

## Materials and methods

**Figure 1:**
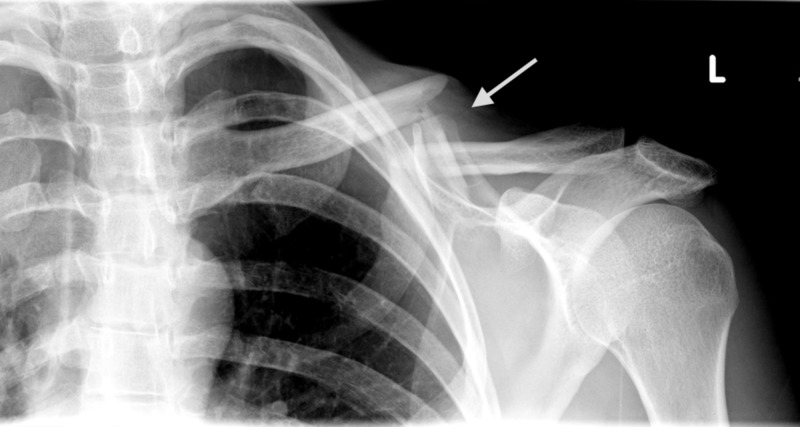
Midshaft clavicle fracture

**Figure 2:**
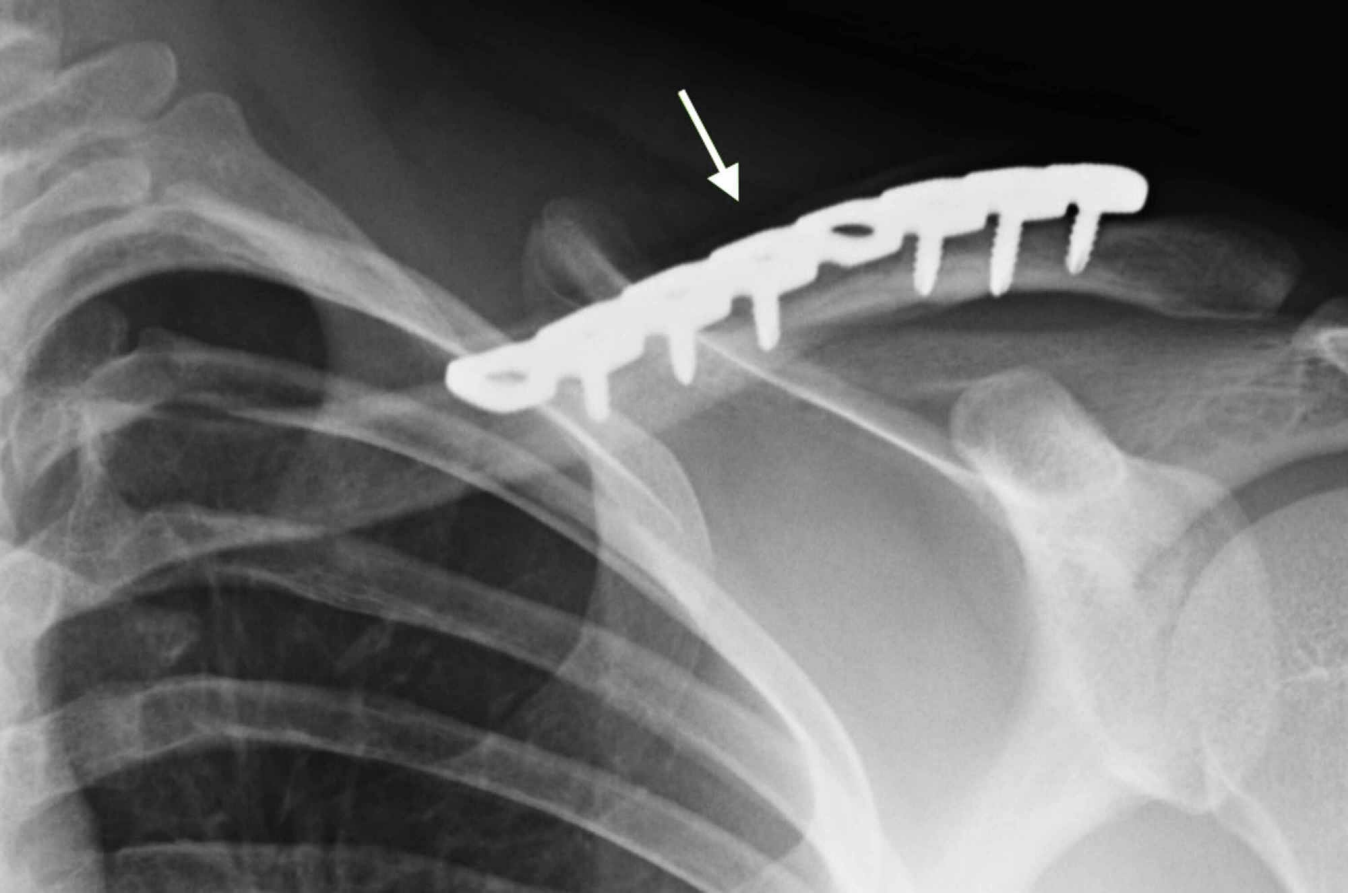
Clavicle operative fixation

We performed a retrospective review of active-duty U.S. Navy and Marine patients with midshaft clavicle fractures treated at Naval Medical Center, San Diego (NMCSD) during the time period Jan 1, 2010 through May 1, 2017 (Figures 1,2). Prior published works on clavicle fractures, including prospective randomized studies, have established a cohort size of 100 patients in the operative and non-operative treatment groups [9]. The selected time frame provided close to or more than 100 patients in each cohort. These patients were identified by a query of medical records for patients with the Current Procedural Terminology (CPT) codes for non-operative treatment of midshaft clavicle fracture (CPT 23500) or open reduction internal fixation of midshaft clavicle fracture (CPT 23515). Patients who were initially treated non-operatively but later underwent surgery were included only in the non-operative group. Inclusion criteria included active-duty military status in the Navy or Marine Corps and treatment at NMCSD for a midshaft clavicle fracture. Average follow-up time from the date of injury or surgery was 28 months. Patients were excluded for open fractures, prior fixation of the clavicle, and lack of any follow-up documentation following treatment. Utilizing chart review, we identified all complications, revision operations, periods of Limited Duty (LIMDU), and need for medical separation from the military following operative and non-operative treatment of a midshaft clavicle fracture. A LIMDU period in the Navy and Marine Corps is a six-month period of time in which a patient is temporarily removed from their regular occupation. This temporary removal from duty allows for recovery from their injury while avoiding physically demanding activities, deployments, shipboard duty, etc. Revision operations were defined as any additional operation in the operative group and any operation after initiating non-operative treatment in the non-operative group. 

We also performed functional outcome analysis on a subgroup of active duty Marines for whom Physical Fitness Test (PFT), and Combat Fitness Test (CFT) data were available. The Marine PFT consists of a three-mile run for time, maximum crunches in a two minute time period, and as many pull-ups as possible without rest. The CFT consists of ammunition (ammo)-can lifts (maximum 30lb overhead lifts in two minutes), a half-mile run for time, and a shuttle run for time. In this subgroup, the Marine Corps Training Information Management System (MCTIMS) database was queried for all marines included in the study. Patients were excluded from this analysis if they had no PFT/CFT data prior to their clavicle injury or if no PFT/CFT data were obtained following their injury or surgery. We evaluated the functional performance of patients by comparing the average number of pull-ups and ammo-can lifts they were able to perform prior to their injury to their scores after their injury and treatment. Our goal was to determine how many patients were able to return to or exceed their pre-injury average at both their next fitness test as well as an additional fitness test at least one year after their treatment course was complete.

Between our two cohorts, rates of infection, non-union, symptomatic malunion, revision surgery, and LIMDU were compared using the Student’s *t*-test. In our functional outcome analysis, we again used the Student’s t-test to compare the rate of patients able to return to or exceed their pre-injury average at two time points. The statistical analysis was performed using the SAS programming language. Statistical significance was defined as *p* ≤ 0.05.

## Results

A total of 269 patients were identified in our CPT code query over a seven-and-a-half-year period. Twenty-two patients were excluded, nine for polytrauma, three for prior surgery, one for open fracture, and nine for having no follow-up data after their initial visit or surgery. Of the 247 patients who met inclusion criteria for our study, 94 patients comprised the operative cohort and 153 comprised the non-operative cohort, with average ages of 28 and 26.6 at the time of injury, respectively. Ninety-three percent of the patients included in the study were male.

Complications included superficial and deep surgical-site infections, non-union, symptomatic malunion, and reoperations, as well as LIMDU periods. The complication rates in each cohort can be seen in Table 1. The rate of infection in the operative group (4%) was significantly greater than in the non-operative group (0%, *p* = 0.023). The rate of non-union in the operative group (3%) was significantly lower than in the non-operative group (14.5%, *p* = 0.004). The rate of symptomatic malunion in the operative group (0%) was significantly different from that in the non-operative group (4.6%, *p* = 0.036). Indications for revision surgery in the operative group included symptomatic hardware removal, deep infection, and non-union, while indications for conversion to surgery in the non-operative group included non-union and symptomatic malunion. There was no statistically significant difference in the rate of revision surgery between the operative (9.2%) and non-operative (13.2%) groups (*p* = 0.105).

**Table 1:** Complication rates for operative vs. non-operative cases of midshaft clavicle fractures Percentage of individuals sustaining each type of complication after injury. * = significant at *p* < 0.05; ** = significant at *p* < 0.005; † Limited Duty

	Operative (n = 94)	Non-Operative (n = 153)
Infection	4.1%*	0%*
Non-Union	3.1%**	14.5%**
Symptomatic Malunion	0%*	4.6%*
Reoperation	9.2%	13.2%
LIMDU †	6.7%	7.8%
>1 LIMDU †	2.3%	2.0%

There was no statistical difference in the rates of patients requiring one or more LIMDU periods for their injury. Finally, there were no patients medically separated for their injury in the non-operative group, while two out of 94 (2.1%) were medically separated in the operative group. This difference was not statistically significant.

In our functional outcome subgroup analysis, a total of 68 Marines were identified from our initial cohorts. Seventeen excluded due to lack of any functional data either before or after their injury. Fifty-one Marines met inclusion criteria, with 22 in the operative cohort and 29 in the non-operative cohort. Of those who underwent operative fixation, 68% were able to meet or surpass their pre-injury average amount of pull-ups in their first PFT after surgery. This number increased to 88% by the next PFT at least one year after surgery. While a similar 69% of non-operative patients met their pre-injury average in their first PFT, only 57% maintained this level at least one year after surgery. The improved performance in the operative group at least one year after their injury was statistically significant (*p* = 0.0468)

Forty-five percent of operative patients were able to meet or surpass their pre-injury average of ammo can lifts in their first CFT after surgery. This number increased to 60% by the next CFT at least one year after surgery. Comparable rates of 60% and 62.5%, respectively, were seen in the non-operative cohort. These differences were not statistically significant. These results can be seen in Table 2.

**Table 2:** Percentage of operative vs. non-operative individuals able to meet or surpass pre-injury fitness test results PFT percentages illustrate the proportion of individuals able to meet or surpass their pre-injury average number of pull-ups at their first PFT after the injury as well as these percentages over a year after injury. CFT percentages illustrate the proportion of individuals able to meet or surpass the number of ammo lifts at each time after injury. * = significant at *p* < 0.05 † Physical Fitness Test; †† Combat Fitness Test

	Operative (n=94)	Non-Operative (n = 153)
1^st^ PFT†	68%	69%
> 1 Year	88%*	57%*
1^st^ CFT††	45%	60%
>1 Year	60%	62.5%

## Discussion

Our study in a military population found comparable union rates and reoperation rates to what has been demonstrated in prior literature on operative and non-operative treatment of midshaft clavicle fractures in both military and civilian patients [13-16]. While our rate of symptomatic malunion in the non-operative group was lower than other recent studies have demonstrated, it was still statistically significantly higher than the rate of 0% in the operative group. There was a similar impact on lost duty time between the treatment groups with regard to LIMDU periods and medical separation. While recent literature suggests earlier return to work following operative treatment, our results may reflect the different demands placed on active duty military in order to return to fit for full duty compared to civilians returning to work [17]. In addition, our study did not capture actual days of work missed but only LIMDU periods needed. As LIMDU periods were needed uncommonly for this injury and may have represented only those patients having a slower recovery, we cannot comment specifically on days of work missed based on our data.

In our functional outcome subgroup analysis, we found that more patients were able to return to or exceed their pre-injury average number of pull-ups at least one year out when treated operatively. The remaining time points and functional tests failed to demonstrate a statistically significant difference between the operative and non-operative groups. While we expected the percentages of patients returning to baseline function to rise as they got further out from surgery, this did not happen in the non-operative group with respect to pull-ups at greater than one year. A follow-up bias may have contributed to this, as there were fewer total marines with testing data at this time point compared to the first post-injury test. Prior results on early and late functional outcomes are mixed. Several studies have demonstrated early superiority in functional outcomes following operative treatment, with improved DASH scores at six and 12 weeks, higher rate of return to moderate activity by two months (80% vs. 50%, respectively) and fewer residual symptoms as far as six months out [14,17]. A prospective cohort study of military patients found superior Constant scores in the operative cohort at all time points out to 18 months [16]. However, a randomized controlled trial by Robinson *et al.* found no significant difference in time to return to work or level of sport between cohorts [9]. These various findings may not be generalizable to all populations. In addition, these results are based nearly entirely on questionnaires.

A recent military review of operatively treated fractures found a 100% union and return to active duty rate. However, they were not able to identify the time to return to duty. Given the unique demands of military service, they called for future studies to evaluate the timing of return to military functional activities [17]. A separate cohort of 28 patients was evaluated for return to military activities such as pushups after surgical fixation via questionnaire. At the mean follow-up of 13 months, 75% reported being able to perform pushups and 21% had deployed [18]. To our knowledge, no study has evaluated performance using physical testing data or compared these results between operative and non-operative cohorts in either a military or civilian population.

Our results are unique in directly evaluating the return to performance of military-specific activities after injury. The finding of significantly improved return to pull-ups in the operative group only beyond one year from injury differs from our original hypothesis. We anticipated that, as suggested by current literature, operative fixation would allow earlier return to function but that these benefits would fade with time. Our findings may be explained by loss of shoulder strength due to malunions in the non-operative group that was not evident until they had time to fully rehabilitate from their injury.

This study has several limitations. First, in regard to sample size, despite a total of 247 patients included, we were only able to obtain fitness data on our current active duty marines. Of this group, 25% were excluded due to insufficient data. Therefore our study may have been underpowered to identify true differences in outcomes. Second, this is a retrospective study without randomization, making our analysis susceptible to bias based on the selection of treatment. Third, a radiographic analysis was outside the scope of this study, which limited our ability to stratify our groups based on fracture severity. Future work on this topic would benefit from including this in our analysis.

## Conclusions

In this study, we were able to use fitness test data to evaluate functional outcomes following operative and non-operative treatment of midshaft clavicle fractures. Our analysis found significantly improved return to function in terms of pull-ups in our operative group compared to our non-operative group at least one year from injury. While similar findings in the literature are based on functional outcome questionnaires, physical fitness performance data has not been reported on. We have a unique opportunity in the military to use this type of tangible functional data to evaluate functional outcomes going forward for this injury and others.

## Appendices

Disclaimer: I am a military service member or employee of the U.S. Government. This work was prepared as part of my official duties. Title 17, U.S.C. §105 provides that copyright protection under this title is not available for any work of the U.S. Government. Title 17, U.S.C. §101 defines a U.S. Government work as work prepared by a military service member or employee of the U.S. Government as part of that person’s official duties. The views expressed in this article are those of the authors and do not necessarily reflect the official policy or position of the Department of the Navy, Department of Defense, nor the U.S. Government.
